# Short Circuit Recognition for Metal Electrorefining Using an Improved Faster R-CNN With Synthetic Infrared Images

**DOI:** 10.3389/fnbot.2021.751037

**Published:** 2021-11-26

**Authors:** Xin Li, Yonggang Li, Renchao Wu, Can Zhou, Hongqiu Zhu

**Affiliations:** School of Automation, Central South University, Changsha, China

**Keywords:** sample synthesis, short circuit detection, infrared image, metal electrorefining, attention-based Faster R-CNN

## Abstract

This paper is concerned with the problem of short circuit detection in infrared image for metal electrorefining with an improved Faster Region-based Convolutional Neural Network (Faster R-CNN). To address the problem of insufficient label data, a framework for automatically generating labeled infrared images is proposed. After discussing factors that affect sample diversity, background, object shape, and gray scale distribution are established as three key variables for synthesis. Raw infrared images without fault are used as backgrounds. By simulating the other two key variables on the background, different classes of objects are synthesized. To improve the detection rate of small scale targets, an attention module is introduced in the network to fuse the semantic segment results of U-Net and the synthetic dataset. In this way, the Faster R-CNN can obtain rich representation ability about small scale object on the infrared images. Strategies of parameter tuning and transfer learning are also applied to improve the detection precision. The detection system trains on only synthetic dataset and tests on actual images. Extensive experiments on different infrared datasets demonstrate the effectiveness of the synthetic methods. The synthetically trained network obtains a mAP of 0.826, and the recall rate of small latent short circuit is superior to that of Faster R-CNN and U-Net, effectively avoiding short-circuit missed detection.

## 1. Introduction

In the metal electrorefining process, short circuits between electrodes cause the temperature of the electrodes to rise, the electrochemical reaction to stop, and the further reduction of the electrolytic efficiency (Aqueveque et al., 2009). Infrared thermography technology has been become a promising method to detect short-circuit electrodes due to its visualization of heat distribution, non-invasive nature, and large-scale monitoring (Maekipaeae et al., 1997; Hong and Wang, 2017). But recognizing short-circuit electrodes from infrared images is still a challenge because of the occlusion above the electrolytic cell group and the complex heat conduction. Temperature of the fault electrode increases, but the canvas on the cell surface may hide the abnormal heat, leading to missed detection. The complex heat conduction between the canvas and the electrodes will interrupt and spread the short-circuit temperature distribution, which will deform the shape of the electrode in infrared image, resulting in inaccurate detection results. In addition, the inherent low resolution of infrared image degrades the detailed features of objects, making it difficult for the electrodes to distinguish and recognize (Xiao et al., 2017; Xing et al., 2019).

With the rise of deep learning, the Convolutional Neural Networks (CNN) have efficiently solved a number of object detection problems by learning more discriminative features (Peng and Chen, 2015; Hiary et al., 2018). CNN based object detection methods are classified into two classes: one-stage detectors like OverFeat (Sermanet et al., 2013), SSD (Liu et al., 2016), and U-Net (Ronneberger, 2017), perform specific classification immediately after feature extract. On the other hand, two-stage detectors such as R-CNN (Girshick et al., 2014), Faster R-CNN (Ren et al., 2017) generate region proposals with low-level cues first and then use the proposals to target existing judgments prior to the classification (Ce et al., 2018). Among recent deep learning methods, Faster R-CNN shows excellent detection performance as Faster R-CNN can capture more pixel-wise annotation information about objects. Also, infrared images usually present poor resolution, low contrast, and fuzzy visual effect, objects in infrared images tend to appear as a series of rough, indistinct areas which closely related to the surrounding complex background. A specific feature and the surrounding area in infrared image both affect the accuracy of target detection. Therefore, in this paper, we use Faster R-CNN model to perform infrared target recognition. However, two challenges are proposed for the task. First, the training of a detection network requires a large number of labeled images, which are labeled with the category and location information of objects in detail (Samadi et al., 2019). Usually such a dataset is collected and annotated manually. It requires the annotator to have expert knowledge of the task and capture the distribution of variables contributing to the varied representation of real world conditions. The laborious work is the first difficulty in deploying CNNs on practical applications. Second, Scale variance enforced by resampling operation in Faster R-CNN may result in information loss, which is even worse for small-scale infrared targets, leading to missed detection, such as the latent short circuit in our problem. Compared to visible images, the features of infrared images degrade, and the convolution layer needs to learn more meaningful features. A complete dataset and a well-designed network are a worthy pursuit.

In order to overcome the difficulty of manually annotating a sufficient number of images and meeting the accuracy requirements of short circuit recognition tasks, we first propose a framework for automatically generating labeled images, and then design an attention-based Faster R-CNN for short circuit detection. In the image synthesis process, we classify short-circuit objects into two categories: obvious short circuit and latent short circuit. Background, target gray scale distribution, and shape are proposed as three key variables after a series of reasonable assumptions and analyses. We simulate the three key variables with different methods to satisfy the diversity of the intra-class of samples. Single cells infrared images that without short circuits are collected as backgrounds; rectangles of random sizes and aspect ratios are exploited to simulate electrode targets; external illumination template and local signal-to-clutter ration (SCR) constraint method are introduced to simulate the multiple manifestations of objects; object locations and class labels are automatically annotated. Then, to increase the detection accurancy, our detection scheme improves the Faster R-CNN by introducing an attention module. This module fuses the semantic segment information of small-scale latent short circuits and the synthetic dataset, making the network focus on small objects during the extraction of features. Combined with anchor parameters fine-tuning and transfer learning strategy the attention-based Faster R-CNN can better avoid latent short circuit missed detection. Summarizing the above discussion, we aim at addressing the problem of automatic synthesis of labeled infrared images and apply it to the training of the short circuit recognition system. The contributions of the paper can be summarized as the following two aspects:

1. Propose an automatic sample synthesis method that can generate a sufficient number of labeled infrared images.
2. Improve the Faster R-CNN by introducing an attention module and design the short circuit recognition system for metal electrolysis, the system is trained only on synthetic samples and generalizes well to real images especially for the latent short circuit class.

The remainder of the paper is organized as follows. Section 2 provides related works about sample number increase method-data augmentation and background knowledge about metal electrorefining. Synthesis difficulties are also discussed in this section. Section 3 presents the details of our synthesis method and the short circuit recognition system based on attention-based Faster R-CNN. Section 4 provides three experiments to evaluate the synthesis method and demonstrate the effectiveness of the system. Finally, the conclusion of our research is presented in Section 5.

## 2. Related Work

### 2.1. Methods About Data Augmentation

Various data augmentation methods have been studied to create additional training data. Generative adversarial networks (GANs) and its variations (Goodfellow et al., 2014; Odena et al., 2016; Zhang et al., 2016) show promising results for highly realistic image generation. The GANs-based method generates images by simultaneously training two models: generative model and discriminative model. But balancing the two models is a difficult task (Ngxande et al., 2019). Another method achieves data augmentation by combining multiple image transformation operations on an existing data set while preserving class labels (Ratner et al., 2017; Gao et al., 2020). For example, flipping, cropping, and color casting are applied to increase the number of marine organism images (Huang et al., 2019). The method mainly imitates the variable elements of the scene that contribute to the samples diversity, such as ambient illumination, target perspective and scale, etc. However, the above augmentations rely on a certain amount of images that have already been labeled. For some specific application scenarios in which the images are difficult to obtain, a third-part public dataset can be exploited, because the content of the dataset has similar features with the application scenario. The optical remote sensing images slices of Google Earth are employed to train a deep model, and then the model is applied for ship detection (Jiang et al., 2019). The handwritten images of MNIST are used to simulate long-rang infrared images, in which dim targets are against background clutters (Fan et al., 2018). Nevertheless, a common dataset whose content is similar to the problem scenario is infrequent.

Actually, realistic-looking is not strictly necessary to train a discriminant algorithm. Key variables of the scene are drive, the diversity of sample, and effectiveness in training a neural network (Mayer et al., 2018). In an image, these key variables are usually related to the visual complexity of the scene and multiple manifestations of objects. Different geometric shapes are adopted to generate targets for machine learning (Silva et al., 2019). Irrelevant pictures are taken as complex backgrounds, and on the backgrounds vehicle license plates are synthesized, then the synthesis images are used for identification training (Björklund et al., 2019). Traditional data augmentation methods may be heavyweight and more expensive in metal electrolysis due to the complexity and uniqueness of infrared scenario. The above research provides valuable references and meaningful inspirations for our problem. The complexity comes from the heat conduction, and the uniqueness is because all cells have a similar structure. Identifying variables about the background and the targets of metal electrolysis infrared image makes sample synthesis possible.

### 2.2. Background of Metal Electrorefining

**Figure 1:**
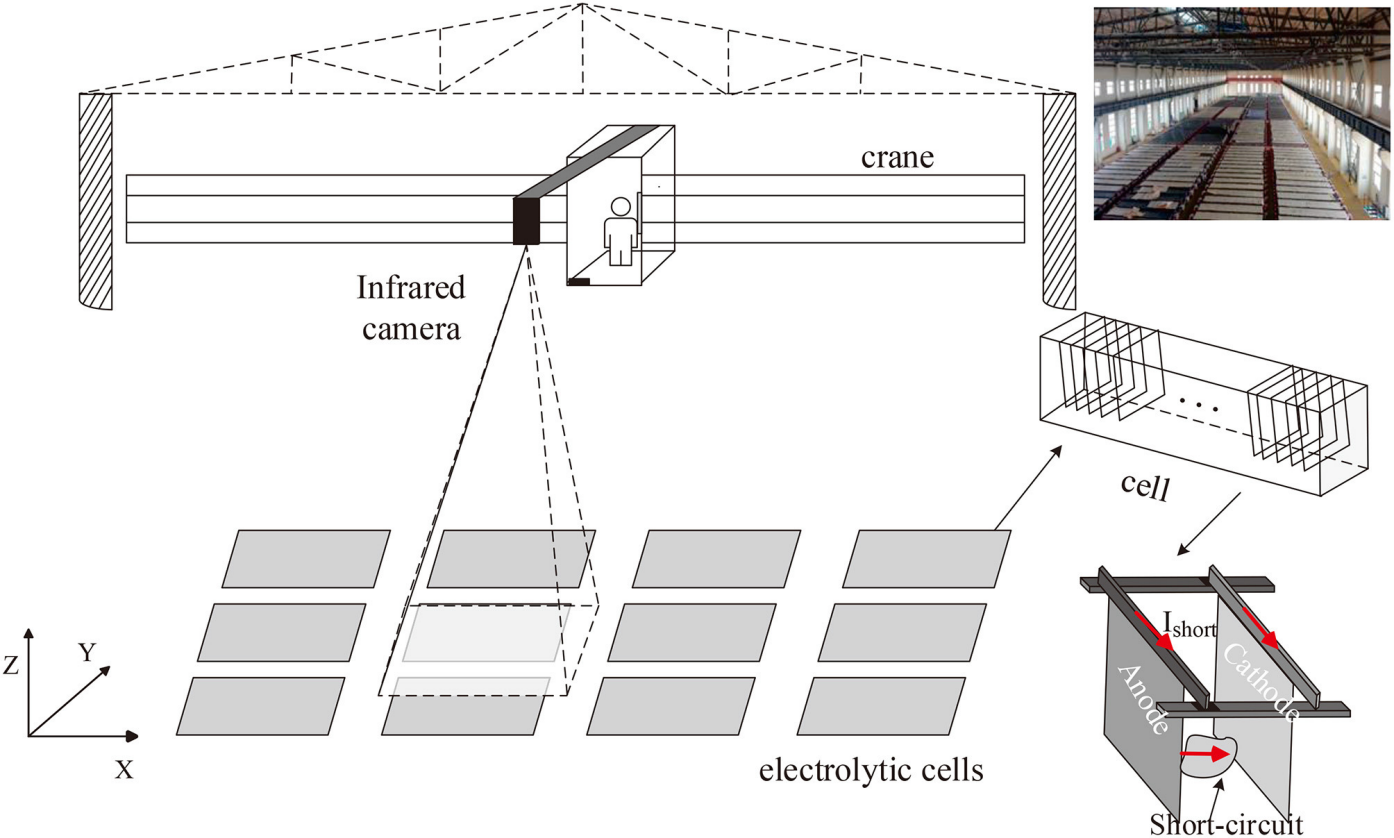
Infrared camera above electrolytic cell groups and the structure of a cell.

**Figure 2:**
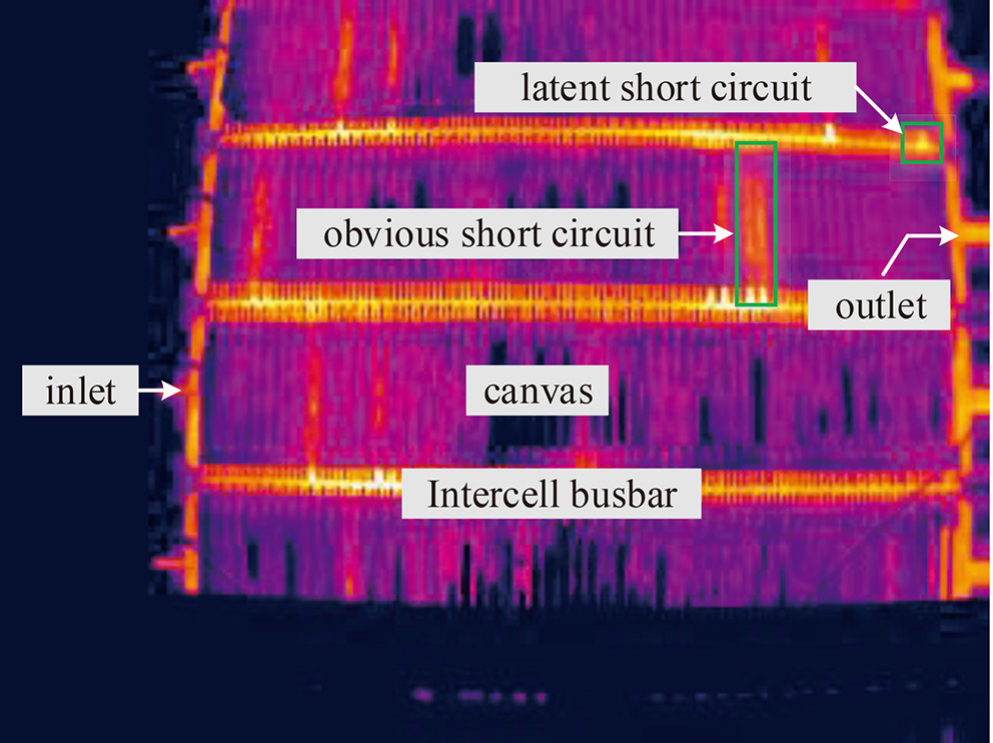
Infrared image containing multiple copper electrolytic cells and different classes of short circuit.

In our problem, take copper electrotrfining as example, the infrared imager is installed on the crane above the electrolytic cell groups to monitor the temperature distribution of electrodes in cells (Figure 1). In each cell, hundreds of electrode plates are parallel immersed into high temperature electrolytes. To prevent electrolyte evaporation, the cell surface is covered with a canvas. In the electrolytic process, the anode is dissolved into metal ions, and then the ions are crystallized out at the cathode plate. Due to the impurities, additive dosing problems, particulates in electrolyte, temperature control, etc. dendrites or nodules growing out from cathode surfaces until they reach anode plates, short circuits occur. The temperature of the short-circuit electrode is obviously higher than that of other electrodes, but the high-temperature electrodes manifest in various forms due to the shielding of the cover. Figure 2 shows an actual infrared image with different types of short circuit and other components of a cell. Other metal electrolysis processes (lead, nickel, etc.) have similar infrared images.

Complexity and randomness of gray scale distribution and the diversity of short-circuit targets pose challenges for infrared image synthesis. Different from realistic visible image synthesis for which the threshold changes of visibility, color appearance, etc. are the most important (Ferwerda et al., 1996), infrared image is the visual result of the thermal distribution of a scene, and it lacks color information. So the gray scale used to reflect the temperature is significant for synthesis.

With canvas, the heat environment in each electrolytic cell is independent, and heat transfer among components of a cell is complex. Thus, the gray distributions of every cell surface are different, although the structure and size of these cells are the same. Also the airflow above cell surfaces adds random attributes to the already complicated background.

Different kinds of short-circuit targets have various manifestations on images, like the short circuits marked in Figure 2. Some short circuits present obvious high gray intensity distribution on the canvas region, and the gray scale distributions are usually not uniform, but they show some common features: the silhouette of the electrode is hazy, the outline appears as a rectangle, and the gray intensity of the positive terminal is obviously higher than other parts. While for some other short circuits, there is no obvious gray scale change on the canvas area, only the end of the electrode which is located on the intercell busbar exhibits high gray intensity. This type of short circuit appears as a small area with uniform gray scale distribution on busbar.

Based on the above analysis, the background, shape, and gray scale distribution patterns are established as three key variables, that are responsible for the large inter-class variability of infrared images. Short-circuits targets are classified into two classes: obvious short circuit and latent short circuit. To determine the positive and negative terminals, we also take the electrolyte inlet and the outlet as another two classes of detection targets that will be annotated on the image (Table 1).

**Table 1:** Target class labeled in the synthesis images.

**Class number**	**Target class**
1	obvious short circuit
2	latent short circuit
3	inlet
4	outlet

## 3. Method

**Figure 3:**
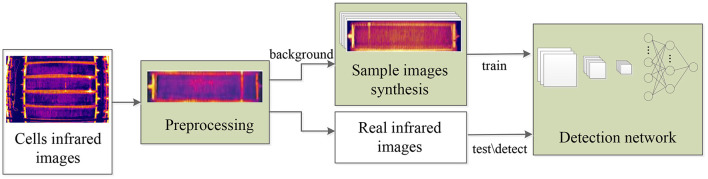
Research route of the work.

The research route of this work is shown as the flow chart in Figure 3. The collected infrared images are firstly corrected for barrel distortion and segmented into individual cells. Then cell images without short circuit are used as background, and we synthesize and label short-circuit targets on it. At last the synthetic image dataset is used for training an improved faster R-CNN detection network to recognize different classes of short circuits, and the network is tested on real world infrared images. Our work mainly focuses on modules of preprocessing, sample images synthesis, and detection network improvement.

### 3.1. Preprocessing of the Infrared Images

Infrared electrolytic cells images suffer from barrel distortion due to the use of the wide-angle lens. So barrel distortion correction is first carried out to facilitate the acquisition of the image patch of a single electrolytic cell.

Distortion occurs because of the inconsistent transmittance of the lens. The refractive index at the edge of the lens is greater than that at the center of the lens. Thus, the same object looks smaller in the outer region of the image than in the central region because the outer region is more compressed than the region near the distorted center. Assume that the distortion rate is radial about the distortion center (Asari et al., 1999), through mapping pixels of the distorted image onto a corrected image, we can obtain the corrected pixel coordinates.

(*x*_*c*_, *y*_*c*_) represents the center of the distorted image, and (*x, y*) are coordinates of any pixel. Radius *r* and the angle θ of a vector from the distortion center to (*x, y*) are given by:


(1a) $$\begin{array}{l} r=\sqrt{{\left(x-x_{c}\right)}^{2}+{\left(y-y_{c}\right)}^{2}} \end{array}$$

(1b) $$\begin{array}{l} \theta =arctan\left(\frac{y-y_{c}}{x-x_{c}}\right) \end{array}$$

The pixel location (*x, y*) in the distorted image can be transformed to a new location (*x*_*new*_, *y*_*new*_) in the corrected image. The corresponding radius *r*_*new*_ and angle θ_*new*_ of the vector from the corrected center (*x*_*nc*_, *y*_*nc*_) to (*x*_*new*_, *y*_*new*_) can be computed as:


(2a) $$\begin{array}{l} r_{new}=\sqrt{{\left(x_{new}-x_{nc}\right)}^{2}+{\left(y_{new}-y_{nc}\right)}^{2}} \end{array}$$

(2b) $$\begin{array}{l} \theta_{new}=\theta \end{array}$$

The mapping relation between radius *r*_*new*_ and *r* is defined with a polynomial as:


(3) $$\begin{array}{l} r_{new}=\overset{n}{\underset{i=0}{\sum }}k_{i}r^{i} \end{array}$$

Where *n* is the number of polynomial terms. *k*_*i*_ denotes the distortion coefficient. The effect of higher order terms can be ignored, because the distortion rate is very small, so the quadratic mapping relationship is adopted. The distortion coefficients can be estimated by fitting the pixel coordinates of the cell boundary in the distorted image In our work, the estimated distortion coefficients are as shown in Table 2.

**Table 2:** Distortion coefficients estimated by fitting pixels on cells border.

**Distortion coefficient**	***k*_0_**	***k*_1_**	***k*_2_**
Value	1	2.355 × 10^−4^	2.285 × 10^−6^

Then, coordinates of the new location (*x*_*new*_, *y*_*new*_) can be obtained by:


(4a) $$\begin{array}{l} x_{new}=x_{nc}+r_{new}cos\theta \end{array}$$

(4b) $$\begin{array}{l} y_{new}=y_{nc}+r_{new}sin\theta \end{array}$$

**Figure 4:**
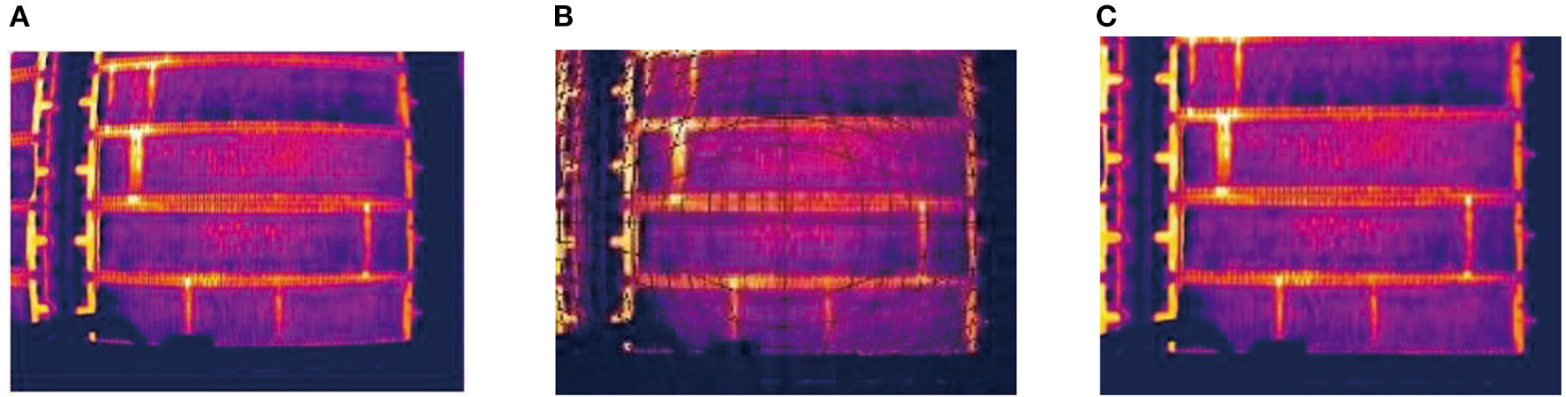
Process of barrel distortion correction: **(A)** A barrel distorted IR image. **(B)** Vacant pixel grid produced by pixel radius mapping. **(C)** The final corrected image after bilinear interpolation.

**Figure 5:**
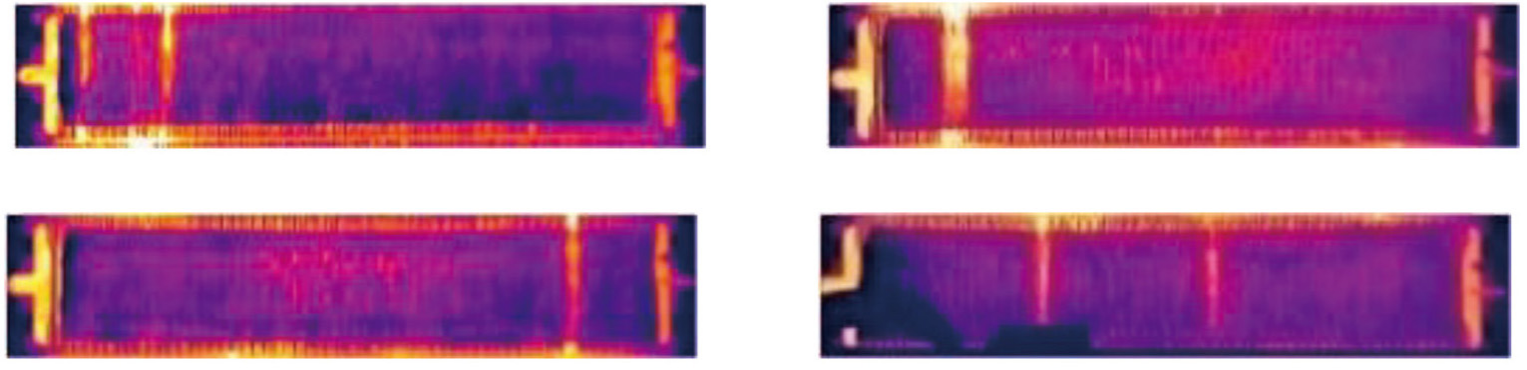
Four single electrolytic cells cropped form the barrel distortion corrected image.

The method corrects the distortion by shifting the pixels. Moving pixels causes vacancy in the original pixel position and thus form a grid of blank pixels on the corrected image. For ease of viewing, we use a black grid in Figure 4B. A bilinear interpolation method is employed to fill these vacant pixels. The final result of distortion correction is as shown in Figure 4C. With the corrected image, we can easily obtain image patches of single cells like in Figure 5.

### 3.2. Sample Images Synthesis

#### 3.2.1. Simulate Background

The background is difficult to simulate because of its complex and random gray scale distribution. That is due to the complex electrochemical reactions in the cell and the heat conduction between the canvas, electrode, and electrolyte. It is unique for the metal electrorefining scene, the alternative of using other backgrounds fails here (Björklund et al., 2019).

**Figure 6:**
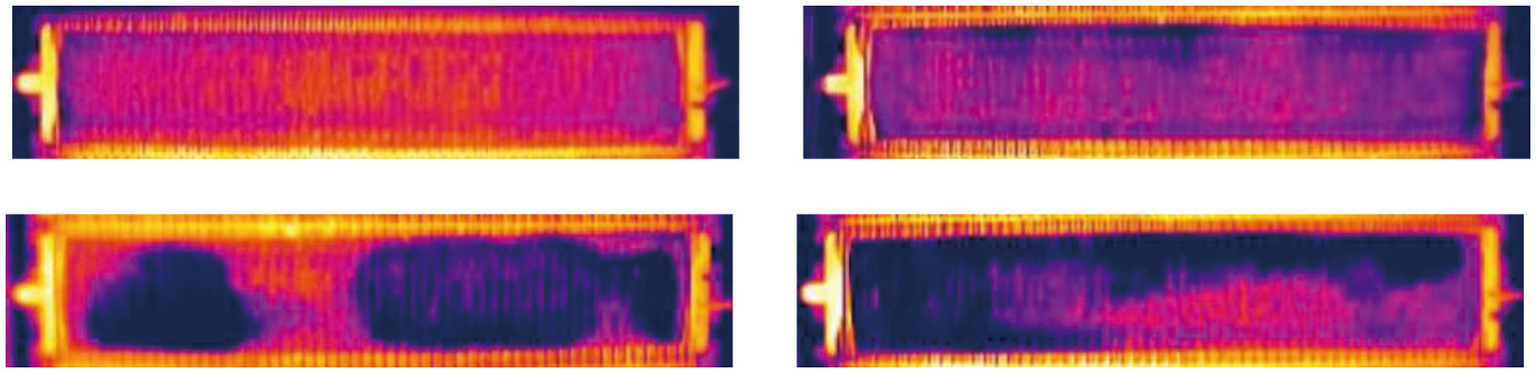
Single cell images without short circuit used as background.

The diversity of the background has a serious influence on target recognition. The complexity and randomness of the gray scale distribution of cells without short circuits gives the image diversity, and such cells can be used as backgrounds. After barrel distortion correction, we can easily obtain any number of single cell images with the same structure. Therefore, we collect enough images of single cells without short circuits as backgrounds (Figure 6) to satisfy the diversity of backgrounds. On these backgrounds, we further synthesize targets.

#### 3.2.2. Simulate Shape

We chose a rectangle to simulate the shape of the electrode. For the obvious short circuit class, although the rod is invisible, the corresponding area on canvas is a strip of high gray intensity. The strip starts from the busbar area and has a larger aspect ratio. For the latent short circuit class, the high intensity area is small and with a small aspect ratio that is approximately 1:1. The latent short circuit is contained within the intercell busbar area. Hence, rectangles with different aspect ratios are used to simulate the shape variable of short-circuit targets.

Electrodes width is calculated by geometric method, and the width range on the image is [4, 13] pixels. Similarly, the length range is [4, 65] pixels. The length and width of the rectangle are randomly selected in the two intervals to construct a rectangle to simulate a short circuit. We set that when the aspect ratio is greater than 1.5, the rectangle is an image patch for obvious short circuit; when the aspect ratio is less than 1.5, the rectangle is an image patch for latent short circuit.

**Figure 7:**
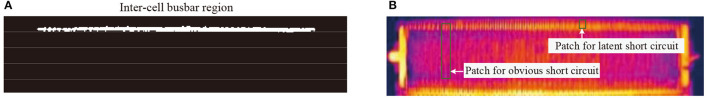
**(A)** Intercell busbar region identified by gray threshold on background image. **(B)** Two example patches for latent short circuit class and obvious short circuit class, the upper-left coordinates of the patches are located in the intercell busbar region.

The location constraint for each target is that the coordinates of the upper-left corner are located within the scope of busbar region. So the intercell busbar needs to be located on the background image first. This can be implemented with a gray scale threshold. The busbar region of Figure 7B is as shown in Figure 7A. In the busbar region, a pixel position is randomly selected, and then the image patch of the target is determined by using the randomly selected size values. It means that the label information (class, location) of a synthetic target is deterministic.

The location information of a target label is expressed as *Patch* = [*x*_*p*_, *y*_*p*_, *w, l*]. (*x*_*p*_, *y*_*p*_), that is the upper-left coordinate. (*w, l*) are the width and length of the image patch. The pixel values of the patches in the raw background image are temporarily reserved for the next gray scale distribution simulation. Two example patches are as shown in Figure 7B.

#### 3.2.3. Simulate Gray Scale Distribution

After, the target patches are determined through assigning gray scale intensity for these patches to simulate the gray scale distribution. Short circuit gray scale intensity is usually higher than the surrounding area, but its distribution is characterized by complexity and diversity. Moreover, the two classes of targets are against different backgrounds. The gray scale continuity of the synthesized target and background should be considered. Therefore, we adopt two different gray scale assignment methods to simulate gray distribution for the two target classes.

For the latent short circuit class, the target should be located in the busbar background with high gray scale intensity and small scale area. The gray scale distribution is smooth. These features make it difficult to distinguish between a latent short circuit and a background. This difficulty can be quantitatively analyzed with SCR (Wang et al., 2017). SCR is a measure of target detectability, and the calculation formula is:


(5) $$\begin{array}{l} SCR=\frac{\left|\mu_{T}-\mu_{B}\right|}{\sigma_{c}} \end{array}$$

Where, μ_*T*_ and μ_*B*_ represent the average intensity of the target and the background, respectively. σ_*c*_ is the standard deviation of the background. |μ_*T*_ − μ_*B*_| is used to evaluate the gray difference between target and background.

The gray level of the target is affected by the surrounding area, and the influence decreases with the increase of distance. So local background (Chen et al., 2013) is more suitable for infrared target simulation. We set the local background area to three times the target area. With a definite SCR value, we can calculate the μ_*T*_ of the target. Define a gray scale enhancement factor *k* as:


(6) $$\begin{array}{l} k=\mu_{T}/Ave_{o} \end{array}$$

**Figure 8:**
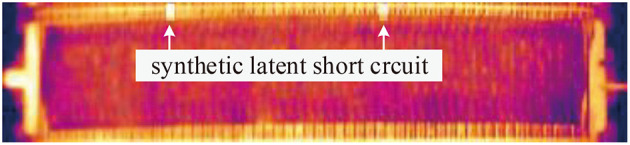
Examples of synthetic latent short circuit instance.

Where, *Ave*_*o*_ is the average intensity of the image patch for latent short circuit that originated from the background image. *K* is multiplied by the pixels in the image patch, the gray value of the image patch increases, and a latent short circuit with a certain SCR value can be obtained. To increase the diversity of samples, the local *SCR* is randomly selected from an interval [1.5, 8] according the research of Kim et al. (2012). This method generates image patch from the infrared background image and preserves the randomness of its gray distribution. Two generated latent short circuit instances are shown in Figure 8.

For the obvious short circuit class, the gray scale distribution is uneven. Affected by random factors, the gray scale distributions are varied. But all the obvious short circuits have one thing in common, that is, there is at least one high gray scale spot in the gray scale distribution. An analogy between the spot and external light source is introduced (Huang et al., 2019). Therefore, a high-intensity spot in the obvious short-circuit area can be regarded as an external light source, affecting adjacent areas. Through adding an additional light source to the image patch for obvious short circuit, we can simulate the diversity of gray scale distribution.

**Figure 9:**
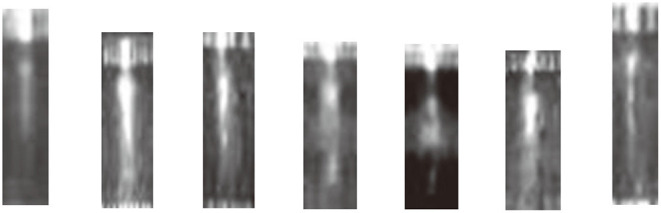
Templates of external light source for obvious short circuit class.

The templates of light source are collected from real infrared images. Some of them are shown in Figure 9. A light source template *O* is randomly selected, then smooth the template with a mean filter as Eq. (7), a gray scale distribution template *E* can be obtained.


(7) $$\begin{array}{l} E=O*X=\frac{1}{MN}\underset{i}{\sum }\underset{j}{\sum }O\left(x-i,y-j\right)X\left(i,j\right) \end{array}$$

Where, *X* is a mean filter, the height *M* and the width *N* of *X* are set 3, and * means the convolution operation.

The size of the synthetic patch for obvious short circuit *P* has been known. Resizing the template *E* to the same size of *P*, we obtained *E*_1_. We calculated the average gray value *Ave*_*E*_1__ of *E*_1_. Then by Eq. (8), the gray difference template *D* was obtained. Through adding pixels (Eq. (9)), the image patch *P* and gray difference template *D* were fused to generate a unique gray distribution for obvious short circuit.


(8) $$\begin{array}{l} D=E_{1}-Ave_{E_{1}} \end{array}$$

(9) $$\begin{array}{l} P_{1}=P+D \end{array}$$

**Figure 10:**
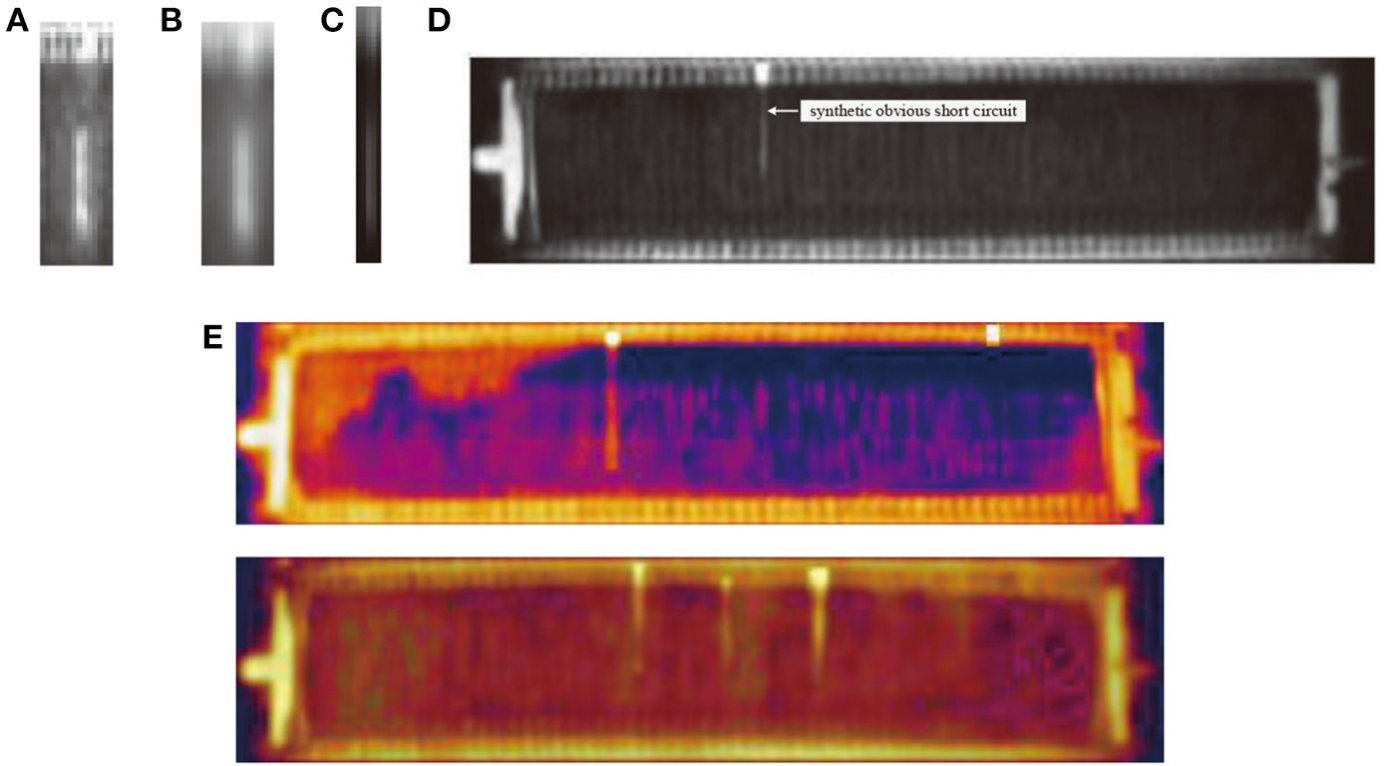
Synthesis process of obvious short circuit class: **(A)** An external light source template. **(B)** Gray scale distribution template after average filtering. **(C)** Difference in template after being resized. **(D)** Synthetic obvious short circuit instance based on the external light source template. **(E)** Examples of synthetic infrared image with obvious short circuit instance and latent short circuit instance.

The above operations strengthened the edge information of the synthetic target. Continuity between the synthesized target and its surrounding area should be maintained in image. Therefore, before adding an external light source, we first reduced the pixel value of *P*. In contrast to the simulation method of latent short circuit gray scale distribution, a smaller SCR value was used to reduce the gray scale. [0.1, 0.3] is an appropriate range obtained by experience. Figure 10 shows the synthesis process of obvious short circuit. The referenced light template is Figure 10A. The synthetic obvious short circuit is generated on the background in Figure 10D. Figure 10E shows some other synthetic images that contain both obvious short circuit and latent short circuit in pseudo color image.

The class of inlet and outlet can be annotated automatically through threshold segmentation or edge detection of the background image, that is not the focus of the work and we will not describe it in this work.

### 3.3. Short Circuit Recognition for Mental Electrorefining With Attention-Based Faster R-CNN

In this section, we first explain the core components of the Faster R-CNN in brief. Then the details of the proposed short circuit detection system with attention-based Faster R-CNN is described. Finally, the strategies of anchor parameters fine-tuning and transfer learning in the system is explained.

#### 3.3.1. Architecture of Faster R-CNN

**Figure 11:**
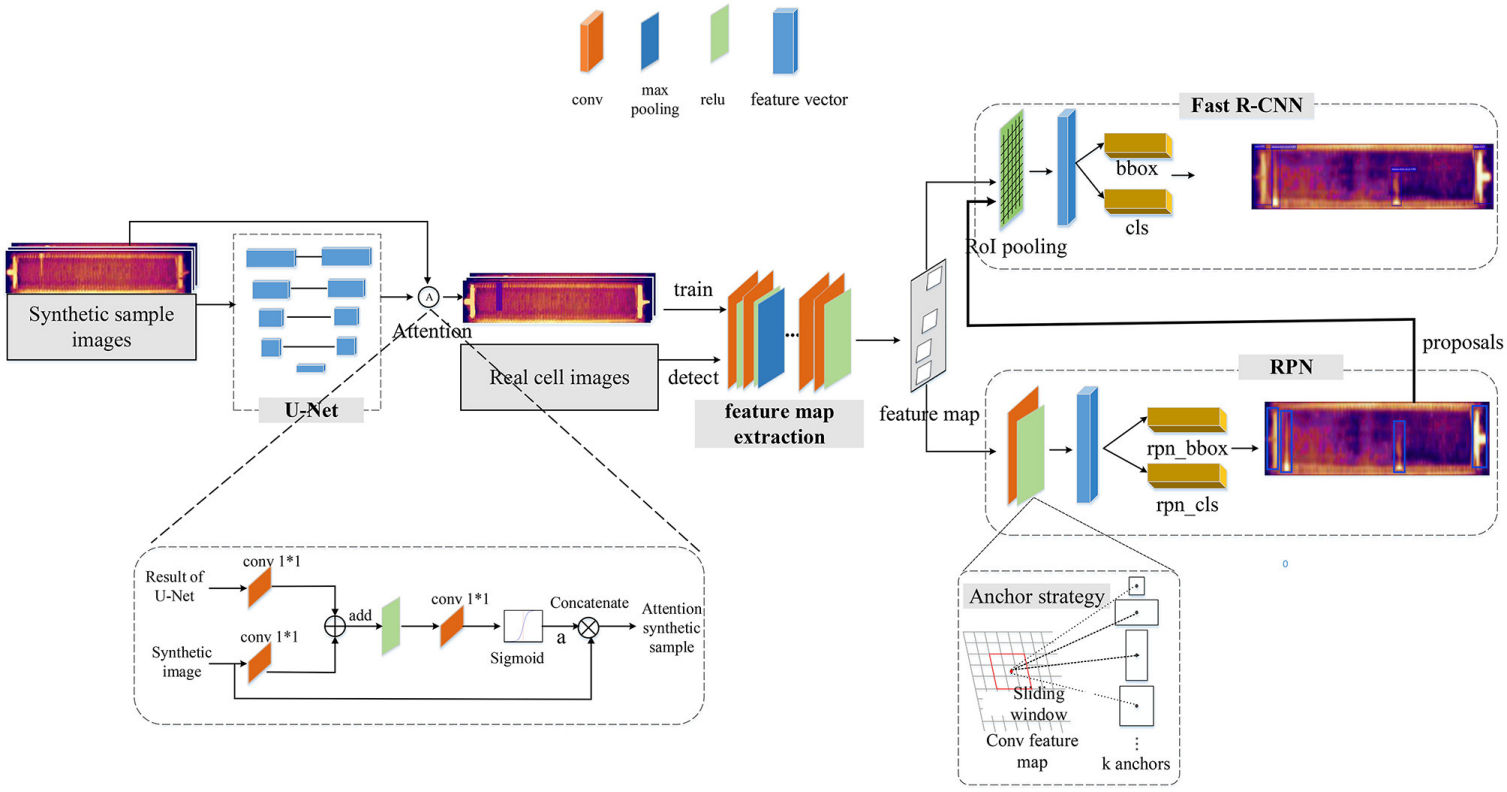
The flowchart of our proposed short-circuit electrode recognition system with attention-based Faster R-CNN.

The Faster R-CNN firstly traverses the feature map of infrared image to distinguish objects from the background irrespective of class, accompanied by bounding box regression to generate region proposals of variable sizes. Then, region proposals are resampled to a fixed-sized box to ensure scale-invariance for categorization. Faster R-CNN consists of three functional components (Figure 11): feature map extraction, region proposal network (RPN) and Fast R-CNN. Convolutional network VGG-16 model (Simonyan and Zisserman, 2014) is used as a backbone to extract feature maps. The extracted feature maps are shared by the RPN and Fast R-CNN. RPN utilize multiscale anchors boxes strategy to generate region proposals from the feature maps. Firstly, the feature maps convolued with a 3 × 3 slide window. Then, on the resulting feature map, nine anchors boxes with 3 basic scale (128^2^, 256^2^, 512^2^) and 3 aspect rations (0.5, 1, 2) are simultaneously generated at each pixel. Feature maps of these anchor boxes are mapped to feature vectors by two 1*1 convolutional kernels, and these feature vectors are used to perform preliminary regression and classification through a fully connected layer. The full connection layers preliminary judge whether there is a target in the anchor box and compute the coordinates of these bounding box. Positive anchor boxes are as recommended region proposals from the input feature maps. Region proposals and the feature maps obtained from convolution layers are fed into Fast R-CNN to perform object classification and bounding box regression.

Although the Faster R-CNN significantly improves detection performance and reduces calculation time through region proposals, it is difficult to detect the small scale targets, like latent short circuits in the infrared images. Because the infrared images lack detailed information, features of small scale latent short circuit are often lost in the sampling process of convolutional networks, which leads to missed detection of the latent short circuit. Compared with false detection, missed detection of the short circuit fault brings greater economic loss and security threat to the electrolytic process.

#### 3.3.2. Attention-Based Faster R-CNN for Small Latent Short Circuit

We introduced attention mechanisms for small scale infrared object detection by combining U-Net and Faster R-CNN. Precisely, the attention module integrate the semantic information of U-Net and the synthetic infrared images to focus on small targets. The architecture is shown in Figure 11. U-Net has a good performance on pixel-wise predictions of small scale objects, so the net is adopted to handle the synthetic images first. U-Net's encoding features and decoding results are skip connected at different scales, realizing pyramid feature fusion and enriching the learned semantic features of small objects. And up sampling restores the edge information of feature image. Thus, the segmentation result of U-Net and the corresponding synthetic images are used as the two inputs of the attention mechanism. The attention module is also given in Figure 11, result of U-Net and synthetic image perform pixel added after 1 × 1 convolution. The result is activated by ReLu and then goes through sigmoid module, the attention coefficient α is obtained. The range of α is [0, 1], if the coefficient is close to 1, the pixel is related to the target characteristics. By concatenating the attention coefficient and the synthetic images, we fuse the semantic information of small objects to the infrared synthetic images. These fused label infrared images then are fed into the Faster R-CNN as the second step of our training procedure.

#### 3.3.3. Anchor Parameters Fine-Tuning for Small Latent Short Circuit

Note that the purpose of using a set of artificially anchored boxes in RPN is to deal with different scales and aspect ratios of objects (Ren et al., 2017). In the original Faster R-CNN, 9 anchor boxes with 3 scales and 3 ratios were used by default. The default basic scales are (128^2^, 256^2^, 512^2^) and the default aspect rations are (0.5, 1, 2). A region proposal is identified by comparing the intersection-over-Union (IoU) overlap of each anchor box with a ground-truth target. The anchor boxes with a high IoU or which satisfy a criterion are assigned as positive.

Such parameter settings may be more applicable to an image datasets, in which targets come in relative large sizes and similar aspect ratios. However, in our application, the sizes of the four kinds of targets are relatively small and the aspect ratios vary greatly. The aspect ratios of latent short circuits are approximate to 1.5, while the aspect ratios of obvious short circuits are usually larger than 2. If the size of the anchor boxes is far larger than that of the ground-truth target, it may lead to any anchor box which can not meet the IoU requirements. Thus, there will be no regional proposal. Furthermore, it causes small-sized targets to fail detection. If the aspect ratio parameter of the anchor boxes are not set properly, the anchor boxes cannot reflect the target shape better, which will also affect the detection accuracy (Sun et al., 2018).

We tuned anchor parameters according to the actual target size. We increase the number of anchors from 9 to 15 by expanding the aspect ratio and reducing the basic size of anchors. The anchors sizes are (8^2^, 16^2^, 32^2^), and five aspect ratios (0.5, 1, 2, 4, 5) are setted. In our problem, this improvement is mainly used to avoid missed detection of the latent short circuit class.

#### 3.3.4. Transfer Learning for Infrared Dataset

Infrared images contain less information compared to visible images, like information about color and texture. Convolutional layers are the most important part to extract feature information by multiple convolutional kernels. Concretely speaking, the shallower convolutional layers can extract lower-level features like edges and hot spots, but deeper layers can extract semantics information that are more important for object recognition. So a transfer learning strategy is adopted. The shallow layers weights of the pre-training model VGG-16 were frozen, and the deep layers weights were retrained. The number of frozen layers was obtained by comparing the results of multiple training. Architecture and the parameter settings of the shared convolutional layers of the VGG-16 are illustrated in Table 3, and the architecture of the RPN and Fast R-CNN are shown in Tables 4, 5, respectively, the content in brackets is the input of the network.

**Table 3:** Transfer learning settings in the feature extractor in Figure 11.

**Layer type**	**Filters**	**Size of kernel**	**Parameter setting**
Input image			
1-1st Conv	64	3 × 3 × 3	Frozen
1-2nd Conv	64	3 × 3 × 64	Frozen
Max pooling layer			
2-1st Conv	128	3 × 3 × 64	Frozen
2-2nd Conv	128	3 × 3 × 128	Frozen
Max pooling layer			
3-1st Conv	256	3 × 3 × 128	Frozen
3-2nd Conv	256	3 × 3 × 256	Trainable
3-3rd Conv	256	3 × 3 × 256	Trainable
Max pooling layer			
4-1st Conv	512	3 × 3 × 256	Trainable
4-2nd Conv	512	3 × 3 × 512	Trainable
4-3rd Conv	512	3 × 3 × 512	Trainable
5-1st Conv	512	3 × 3 × 512	Trainable
5-2nd Conv	512	3 × 3 × 512	Trainable
5-3rd Conv	512	3 × 3 × 512	Trainable

**Table 4:** Architecture of the RPN in Figure 11.

**Layer type**	**Filters**	**Size of kernel**
[5-3rd Conv]Input layer		
RPN Conv	512	3 × 3 × 512
Classification convolutional layer(softmax)	30	1 × 1 × 512
[RPN Conv]Regression convolutional layer	60	1 × 1 × 512

**Table 5:** Architecture of the classifier in Fast R-CNN in Figure 11.

**Layer type**	**Size of output**
[5-3rd Conv] [RPN proposal region]Input layer	
RoI Pooling	7 × 7 × 512 × 32
1st fully connected layer	4, 096 × 32
2nd fully connected layer	4, 096 × 32
Classification fully connected layer(softmax)	32 × 5
[2nd fully connected layer]Regression convolutional layer	32 × 16

## 4. Experiment

In this section, evaluation metrics are introduced first. Then we conduct three experiments. In the first experiment of section 4.2, with a variable-controlling approach we study how each variable of synthesis affects the detection performance of the neural network and verify the effectiveness of the proposed sample synthesis method. In the second experiment of section 4.3, different synthesis methods are compared. Finally, the comparison of original Faster R-CNN (Ori-Faster R-CNN) v.s. Faster R-CNN with anchor parameter fine-tuning (Fin-Faster R-CNN) v.s. Attention-based Faster R-CNN (Att-Faster R-CNN) v.s. U-Net is in section 4.4, also, the short circuit detection result are showed. All the experiments were trained on synthetic samples and texted on actual images.

### 4.1. Quantitative Metrics

Precision-recall (PR) curves of four classes of objects are used for result evaluation. The curve plots the precision against the recall rate of a detector, and it is a visual representation of an algorithm's performance. A detector with a higher precision and a higher recall rate indicates a better discrimination ability. Precision and recall rate are defined as follows:


(10a) $$\begin{array}{l} Precision=tp/\left(tp+fp\right) \end{array}$$

(10b) $$\begin{array}{l} Recallrate=tp/\left(tp+fn\right) \end{array}$$

Where *tp* represents the number of true positives, and *fp* represents the number of false positives. *fn* denotes the number of false negatives. Positive data and negative data mean the four types of detection objects and background region, respectively. A false positive case refers to the case where background is mistaken as a target or one kind of target is mistaken as another kind. A false negative case refers to the case where true positive data is error detected.

Mean Average Precision (mAP) of different classes of objects is used for result comparison. A mAP score is the mean of the average precision (Ap) for each class. The definition of AP is defined as:


(11) $$\begin{array}{l} AP=\int_{0}^{1}p\left(r\right)dr \end{array}$$

Where, *p* is the precision rate, and *r* is the recall rate. In our work, mAP score is reported using an intersection-over-union (IoU) threshold at 0.65. The bigger the mAP score, the better the detection result.

### 4.2. Key Variables Assessment for Synthesis

The significance of the background for target recognition has been studied in visual images, while the other two key variables (variable 1:shape and variable 2:gray scale distribution) effects on synthetic data are less understood. We designed a similar ablation experiment to study how each key variable influenced the synthesis process. The activation ratio of key variables in the dataset was controlled, and then the dataset was used to train the Faster R-CNN. The concept of activation here means that in a training set, the key variable of a specific proportion of samples satisfy diversity, and the variable of the remaining samples are kept constant. Through comparing the effects of different datasets on detection and recognition performance of Faster R-CNN, we analyze the robustness of the key variables.

We first generated an infrared cell image dataset (ICID) as a reference dataset using the procedure we described in section 3.2. ICID contains 1,4257 synthetic images (resolution≈ 70 × 280) and 4 categories of targets: inlet, outlet, obvious short circuit, latent short circuit. We also generated extra 8 annotated datasets where each key variable was active for 30, 50, 70, and 90% of the images, respectively, the numbers of samples are in parentheses in Table 6. To prove the effectiveness of the synthetic sample, we added 200 hand-annotated real images into the ICID to form a new dataset. The 10 datasets were fed to Faster R-CNN, respectively, and then the network tested on realistic electrolytic cells images to recognize the targets.

**Table 6:** mAP score of the Faster R-CNN trained on synthetic datasets with different key variable activation ratio.

**Datasets with key variable activated**	**Activation ratio**			
**key variable activated**	**30% activated**	**50% activated**	**70% activated**	**90% activated**
Variable 1	0.556 (15,649)	0.582 (15,640)	0.585 (14,578)	0.592(14,624)
Variable 2	0.578 (14,520)	0.617 (14,481)	0.619 (14,500)	0.621 (14,510)
ICID (14,257)	0.632			
ICID+Real images (14,457)	0.878			

Table 6 shows the mAP scores of the Faster R-CNN trained on the 10 datasets. Reducing the activation ratio of the two key variables in dataset yields dramatic performance drop, which illustrates the significant impact of the two key variables toward the networks feature learning ability. But when more than 50% of the samples meet the diversity of each key variable, the growth value of mAP increases slowly. When 50% of the images are considered about the shape diversity (variable 1), the score is reduced by 8% compared to the reference dataset ICID; while for gray scale distribution (variable 2), the score is reduced only by 2%. It suggests that the networks learning is obviously more sensitive to target shape change, But the gray scale distributions have a greater effect on learning than target shape, as the absolute value of the increase is larger. Especially when more samples meet the diversity of gray scale distribution, the mAP is close to that of ICID. It means that the key variables of the goal we defined are accurate, After training on ICID, a mAP score of 0.632 is obtained. The score increases to 0.878 when real images are added in. It suggests that the proposed synthetic method is effective and available at the beginning for deploying CNNs on practice metal scenario. The synthetic method helps avoid the laborious work of manually annotating large numbers of images.

**Figure 12:**
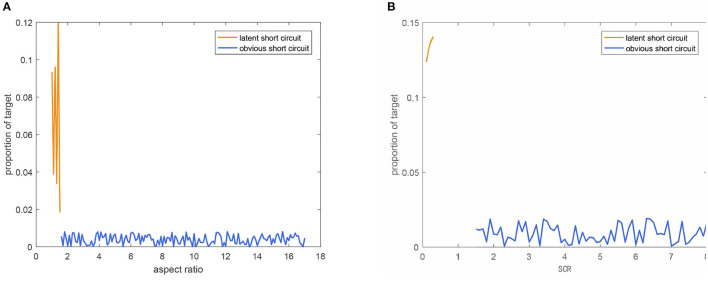
**(A)** Distribution of the two types short circuits with respect to aspect ratio. **(B)** Distribution of the two types short circuits with respect to SCR value.

Figure 12 shows the distribution of the two types of short circuits with respect to aspect ratio and SCR values in ICID. As aspect ratio determines the diversity of key variable 1, and similarly, SCR value determines the diversity of key variable 2 in our proposed synthetic method. Obvious short circuit class accounts for 60% of synthetic target number; the range of aspect ratio and SCR of obvious short circuit class are both obvious wider than latent short circuit, and the distribution is uniform.This indicates that the diversity of samples is guaranteed by a wide range of key variable values and uniform distribution of sample numbers. This is in accord with the actual engineering case.

### 4.3. Comparison of Data Synthesis Method

In this section, in order to compare the performance of different synthesis methods, Faster R-CNN trained on 8 training sets that generated with different methods listed below and tested on real cell images. We manually annotated 200 real images and used the four commonly methods M1, M2, M3, and M4 to augment the labeled images respectively. In M1, random visible light images were used to replace the electrolytic cell background while preserving targets. The set ICID was generated automatically without the aid of any labeled images by using our proposed method (OM).

- M1:Random background (Björklund et al., 2019)
- M2:Flipping
- M3:Color casting
- M4:Noise
- Fusion1: Fusing M2, M3 and M4.
- OM: ICID generated by the propsed synthetic method
- Fusion2: Fusing ICID and M2, M3 and M4.
- Baseline: 200 real image.

Table 7 shows the short circuit detection and recognition results with different data augmentation methods. Compared with the Baseline, all methods improve the performance of short circuit detection except M1. That is because the backgrounds of short circuits are cell surfaces with complex thermal distribution. There is spatial continuity and gray correlation between targets and backgrounds, which can not be learned from other backgrounds. M2, M3, M4, and Fusion1 improve the mAP by 17.1%,4.75% 12.3%, and 31.1%, while the number improved by OM is 58.9%. Fusing the method OM with M2, M3, and M4 does not make a significant difference in detection performance, like the values with the gray shade in Table 7. Compared with other methods, our proposed synthesis method does not rely on any pre-annotated images at all, which is our original intention, that is, to solve the problem of lack of engineering samples effectively and labor-saving. At the same time, we note that in the table, the network's detection performance for latent short circuit has been kept at a low level. That is the subject to be discussed in our next experiment.

**Table 7:** Test results of different data sugmentation methods.

**Methods**	**mAP**	**Obvious short circuit**	**Latent short circuit**	**Inlet**	**Outlet**
M1	0.295	0.313	0.002	0.428	0.437
M2	0.493	0.606	0.006	0.679	0.680
M3	0.423	0.427	0.005	0.623	0.635
M4	0.473	0.613	0.006	0.628	0.646
Fusion1	0.552	0.620	0.007	0.791	0.790
OM	0.669	0.871	0.007	0.891	0.905
Fusion2	0.672	0.883	0.007	0.890	0.907
Baseline	0.421	0.425	0.006	0.621	0.633

### 4.4. Accuracy of the Short Circuit Detection System

In this part, we verify our proposed short circuit detection system with attention-based Faster R-CNN(Att-Faster R-CNN). The method proposed in this paper can effectively improve the recall rate of latent short circuit while still maintaining a relative high detection accuracy about obvious short circuit class. To prove the advantage of our algorithm, we compared it with original Faster R-CNN (Ori-Faster R-CNN), Faster R-CNN with anchor parameter fine-tuning (Fin-Faster R-CNN), and U-Net. All experiments were performed under the same environment.

We evaluated the performance by training all models on ICID and tested on real cell infrared images. In the fine-tuning strategy, instead of 9 anchors with a basic size of 128 × 128, 256 × 256 and 512 × 512 and 3 aspect rations (0.5, 1, 2) used for the PRN (Ren et al., 2017), we increased the number of anchors to 15 referring to the settings of Kim et al. (2018). The anchors sizes are 8 × 8, 16 × 16 and 32 × 32 and the five aspect ratios include 5 : 1, 4 : 1, 2 : 1, 1 : 1, and 1 : 1.5. In the Fast R-CNN classification part, an RoI is treated as foreground with the threshold of *IoU* = 0.65. This choice respects the need for precise fault location in the engineering field. Transfer learning strategy is illustrated in Table 3, the weights of the first five layers from VGG-16 were frozen, and the rest of the layers were retrained. PR curves and RoC curves are adopted to illustrate the detection performance. Sum losses of the four class objects are used to indicate the efficiency of transfer learning.

**Figure 13:**
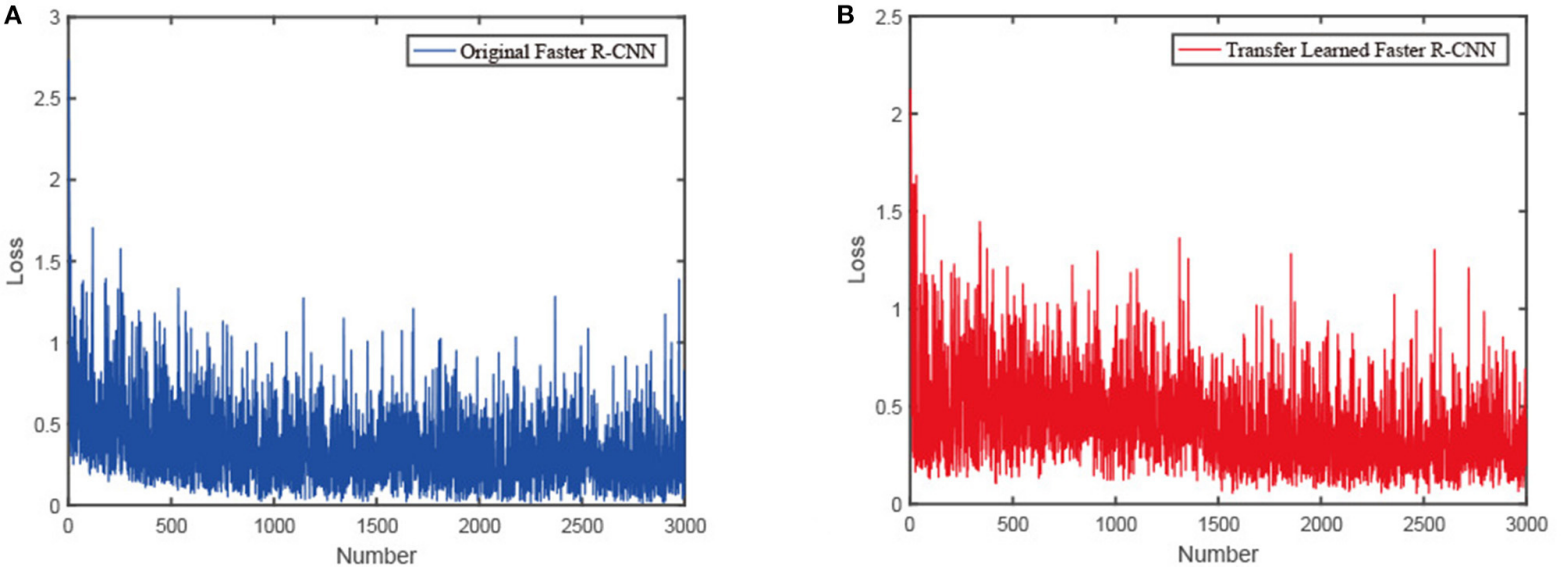
The influence of transfer learning on the training of the Faster R-CNN: **(A)** Loss of the original Faster R-CNN with number of iterations. **(B)** Loss of the transfer learned Faster R-CNN with number of iterations.

Figure 13 shows the learning loss of the Faster R-CNN with and without transfer learning. The loss curve of the original Faster R-CNN converges around 1000 iterations, while the loss curve of the transfer learned Faster R-CNN converges at around 1500 iterations. At the beginning of the training, the gradient descent of our network is slow but stable, whereas the gradient of the original network soon drops to a stable value. It reveals that the transfer learned network can learn more general characteristic information from the dataset.

**Figure 14:**
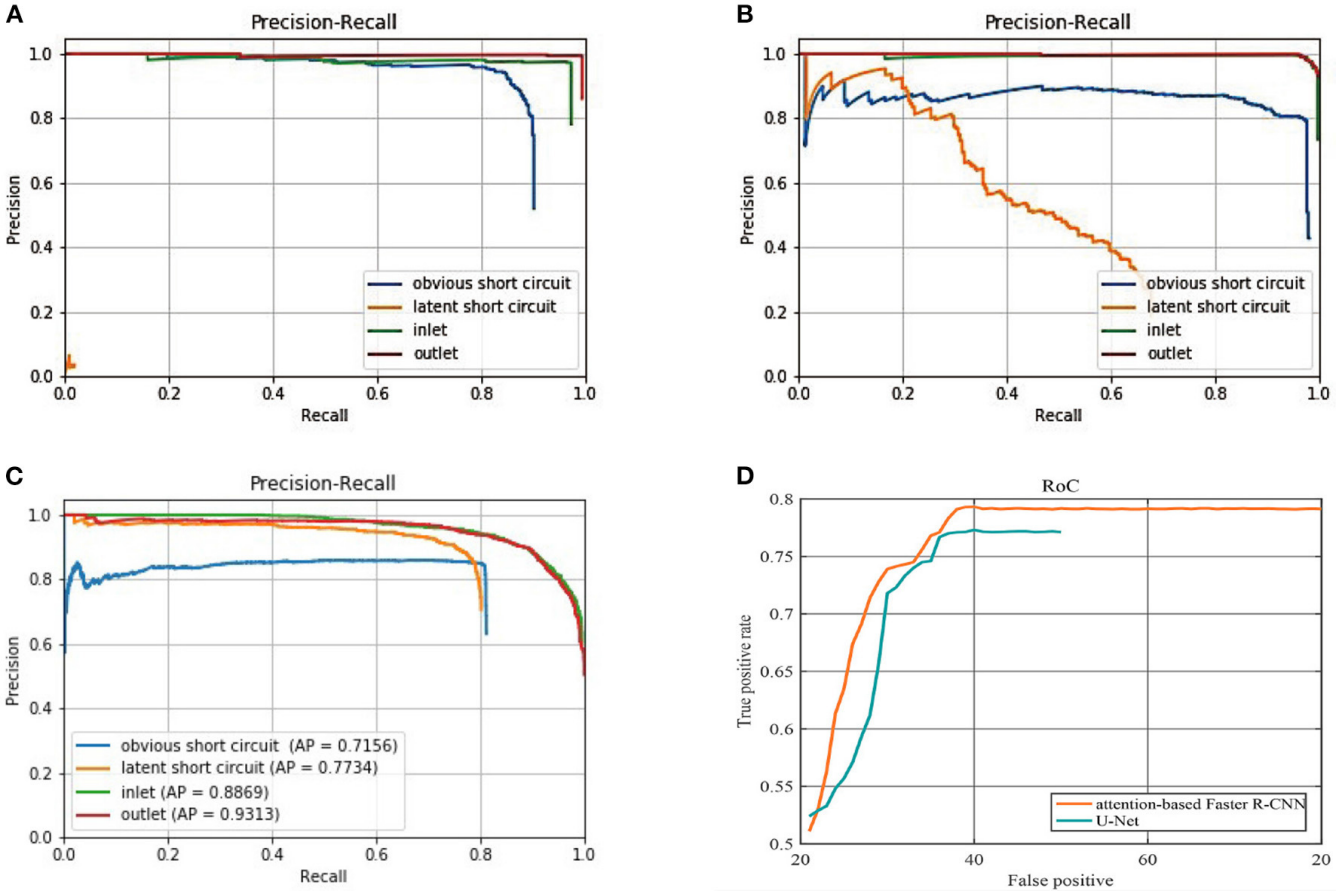
**(A)** PR curves of the Ori-Faster R-CNN. **(B)** PR curves of the Fin-Faster R-CNN. **(C)** PR curves of the Att-Faster R-CNN. **(D)** RoC curves of Att-Faster R-CNN and U-Net for latent short circuit detection, on the x-coordinate, we replaced FPR with total false positive number.

Figures 14A–C presents the detailed comparisons of three sets of PR curve for the four classes of objects, respectively with Ori-Faster R-CNN, Fin-Faster R-CNN, and Att-Faster R-CNN. Furthermore, comparision of small latent short circuit detection results with Att-Faster R-CNN and U-Net is also provided in Figure 14D. In conjunction with Figure 14 and Table 8 it can be observed that all models show the best detection performance on classes of inlet and outlet. Fin-Faster R-CNN can alleviate the difficult detection problems of latent short circuit, but keep a high recall rate need to sacrifice the precision. It is a dilemma for engineering management. Att-Faster R-CNN shows the best detection performance for latent short circuit. Because the attention mechanism integrates more semantic information about latent short circuits into the synthetic sample, aided by the variable anchor scale, the Att-Faster R-CNN can show a stable high precision at a high recall rate. Fin-Faster R-CNN and Att-Faster R-CNN both exhibit precision decline for obvious short circuit class. It is an acceptable decrease of accuracy under the premise that miss detection of short circuits has a more serious impact on production. In Figure 14D, reliability of Att-Faster R-CNN is superior to U-Net, The promising results validate the effectiveness of the proposed attention mechanism for latent short circuit detection.

**Table 8:** The four class of targets average precision of Faster R-CNN with different anchor parameter setting.

**Method**	**Obvious short circuit**	**Latent short circuit**	**Inlet**	**Outlet**	**mAP**
Ori-Faster R-CNN	0.872	0.006	0.898	0.907	0.671
Fin-Faster R-CNN	0.815	0.460	0.907	0.908	0.772
Att-Faster R-CNN	0.715	0.773	0.887	0.931	0.826
U-Net	–	0.752	–	–	–

**Figure 15:**
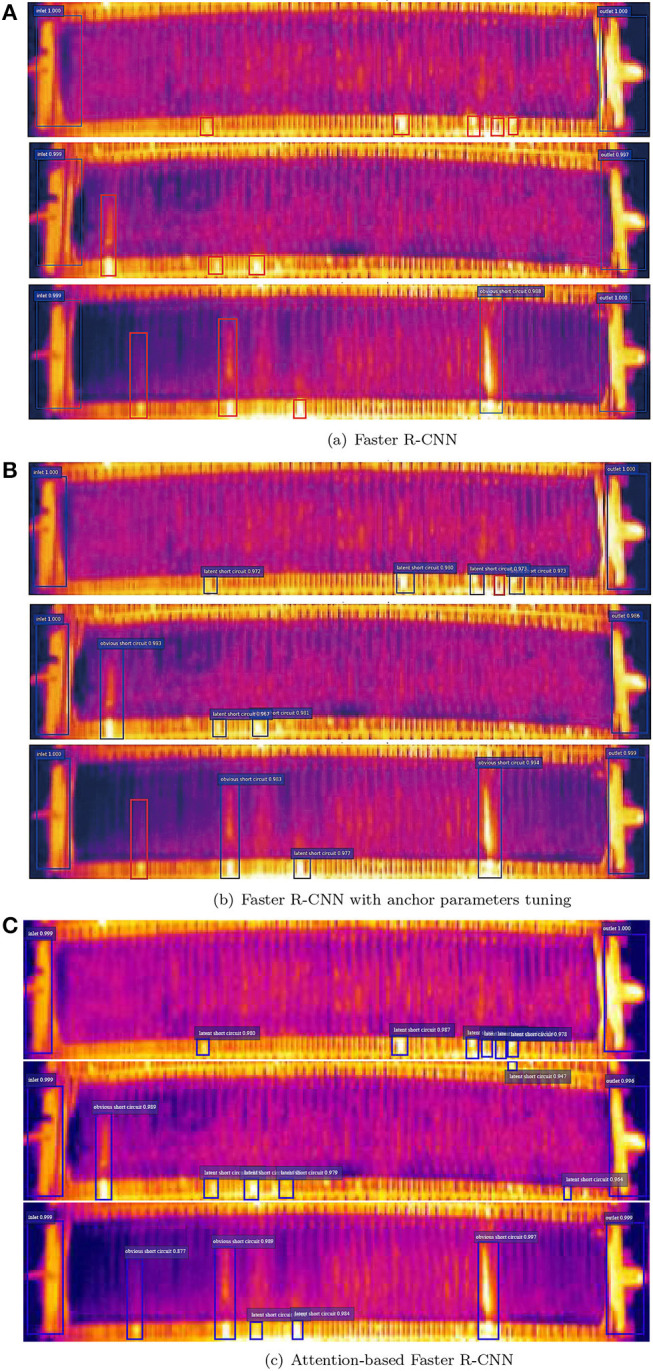
**(A)** Short circuit detection result with original Faster R-CNN. **(B)** Short circuit detection result with Faster R-CNN with anchor parameter fine-tuning. **(C)** Short circuit detection result with attention-based Faster R-CNN.

Figure 15 illustrates the detection results with the three Faster R-CNN network in three same scene, respectively. Attention-based Faster R-CNN able to accurately detect more latent short circuit. Whereas, the result with Ori-Faster R-CNN and Fin-Faster R-CNN lists more false negatives in the red box.

## 5. Conclusions

This work focused on short circuit detection in infrared image of metal electrolysis scene with CNNs. An infrared image synthetic method is proposed to automatically generate labeled infrared dataset ICID by simulating key variables of the scenario that affect the diversity of samples. Additionally, attention-based Faster R-CNN is proposed and used to design the short circuit detection system. In the system, an attention module integrates the semantic segment results of U-Net with the synthetic ICID to obtain rich representation ability on the infrared images. Combined with strategies of anchor parameters fine-tuning and transfer learning, the detection system can efficiently avoid the missed detection of a latent short circuit, and the performance is superior to the original Faster R-CNN and U-Net. The proposed method is specifically dedicated to metal electrolysis scenes, but the methodology of mining targets' key variables to automatically synthesize samples will be further extended to other application areas and training algorithms.

## Data Availability Statement

The raw data supporting the conclusions of this article will be made available by the authors, without undue reservation.

## Author Contributions

XL: programming and preparation, creation and writing the initial draft. YL: review and editing, providing study materials. RW: programming. CZ: design of methodology. HZ: formulate research goals and aims. All authors contributed to the article and approved the submitted version.

## Funding

This work was supported by the National Key R&D Program of China (grant no. 2019YFB1704700), the National Natural Science Foundation of China (grant no. 61890930-2), and the Postgraduate Research Innovation Project of Central South University (grant no. 2018zzts174).

## Conflict of Interest

The authors declare that the research was conducted in the absence of any commercial or financial relationships that could be construed as a potential conflict of interest.

## Publisher's Note

All claims expressed in this article are solely those of the authors and do not necessarily represent those of their affiliated organizations, or those of the publisher, the editors and the reviewers. Any product that may be evaluated in this article, or claim that may be made by its manufacturer, is not guaranteed or endorsed by the publisher.
